# Recyclable Keggin Heteropolyacids as an Environmentally Benign Catalyst for the Synthesis of New 2-Benzoylamino-*N*-phenyl-benzamide Derivatives under Microwave Irradiations at Solvent-Free Conditions and the Evaluation of Biological Activity

**DOI:** 10.3390/molecules23010008

**Published:** 2017-12-21

**Authors:** Karima Ighilahriz-Boubchir, Baya Boutemeur-Kheddis, Cherifa Rabia, Malika Makhloufi-Chebli, Maamar Hamdi, Artur M. S. Silva

**Affiliations:** 1 Laboratoire de Chimie Organique Appliquée (Equipe Hétérocycles), Faculté de Chimie, Université des Sciences et de la Technologie Houari Boumediène, BP 32, El-Alia, Bab-Ezzouar 16111, Algeria; karima_ighil@yahoo.fr (K.I.-B.); makhloufi_malika@yahoo.fr (M.M.-C.); prhamdi@gmail.com (M.H.); 2 Laboratoire de Physique et Chimie des Matériaux (LPCM), Université Mouloud Mammeri, BP17RP, Tizi Ouzou 15000, Algeria; 3 Laboratoire de Chimie du Gaz Naturel, Faculté de Chimie, Université des Sciences et de la Technologie Houari Boumediène, BP 32, El-Alia, Bab-Ezzouar 16111, Algeria; c_rabia@yahoo.fr; 4 Department of Chemistry & QOPNA, University of Aveiro, 3810-193 Aveiro, Portugal; artur.silva@ua.pt

**Keywords:** Keggin-type heteropolyacids, 2-benzoylamino-*N*-phenyl-benzamide derivatives, microwave irradiation, solvent free conditions, antibacterial, antifungal

## Abstract

2-Benzoylamino-*N*-phenyl-benzamide derivatives (**5a**–**h**) were prepared from 2-phenyl-3,1-(4*H*)-benzoxazin-4-one **3** and substituted anilines **4a**–**h** in the presence of a Keggin-type heteropolyacids series (H_3_PW_12_O_40_·13H_2_O; H_4_SiW_12_O_40_·13H_2_O; H_4_SiMo_12_O_40_·13H_2_O; and H_3_PMo_12_O_40_·13H_2_O) as catalysts without solvent and under microwave irradiation. We found that the use of H_3_PW_12_O_40_·13H_2_O acid coupled to microwave irradiation allowed obtaining a high-yielding reaction with a short time. The compound structures were established by ^1^H-NMR and ^13^C-NMR. The antibacterial and antifungal activities of the synthesized compounds exhibited an inhibition of the growth of bacteria and fungi.

## 1. Introduction

The concept of the green chemistry consists in the development of an environmentally friendly approach for organic synthesis using ecological and efficient protocols [1]. In order to develop a methodology that could fit into the green chemistry field, for the synthesis of new 2-benzoylamino-*N*-phenylbenzamide derivatives via benzoxazinone, the choice was made on the use of bothpolyoxometalates (POMs) as catalysts, known for their efficiency, and microwave irradiation for time-saving.

Benzoxazinones can be used as precursor for the synthesis of wide variety of heterocyclic compounds, such as quinazolinones and quinazolines [2,3,4]. The benzoxazinone derivatives are already known for their biological and pharmacological activities [5,6], as anti-convulsants [7,8,9], antihypertensive [10], analgesic [11,12], anti-inflammatory [13],antimicrobial [14,15,16], antifungal [17,18] andantibacterial [19] activities, antimuscular contractor and hypnotic activities [20], anti-fetal activity [21], antidiabetic and hypolipidemic activity [22], and as antidepressants [23]. The benzoxazinones were also tested for their inhibitory activity toward human leukocyte elastase [24,25], antimalarial, anticancer, and anti-HIV [26,27].

As benzoxazinones, the 2-benzoylamino-*N*-phenylbenzamide derivatives can be also used as precursors for both quinazolinone and quinazoline synthesis, and can also present biological and pharmacological activities.

The POMs, particularly the heteropolyacids (HPAs), having the Keggin structure, have received much attention for organic synthesis. They are soluble in all the solvents, which allows for the recovery of the synthesized product by simple filtration [28]. Thus, HPAs offer a strong option for efficient and cleaner processes compared to polluting and corrosive liquid acid catalysts, such as mineral acids. Effectively, in previous works, HPAs showed excellent catalytic activities in several reactions as the synthesis of substituted 1,4-diazepines and 1,5-benzodiazepines [29], 4(3*H*)-quinazolinones [30], calix [4] resorcinarenes [31], and 3,4-dihydropyrimidinones [32].

Among the derivatives of the 2-benzoylamino–*N*-phenylbenzamide (**5a**–**h**) series, 2-benzoylamino-*N*-phenylbenzamide **5a** was synthesized from 2-phenyl-1,3-(4*H*)–benzoxazin-4-one **3** and aniline in the presence of HPAs series as formula H_3_PW_12_O_40_ (PW_12_), H_4_SiW_12_O_40_ (SiW_12_), H_3_PMo_12_O_40_ (PMo_12_) and H_4_SiMo_12_O_40_ (SiMo_12_), under microwave irradiation and solvent-free conditions. Then, the most efficient catalyst was used to synthesize all the series of 2-benzoylamino-*N*-phenylbenzamide derivatives via benzoxazinone **3**, in the presence of substituted anilines (**4a**–**h**).

## 2. Results and Discussion

**Scheme 1:**
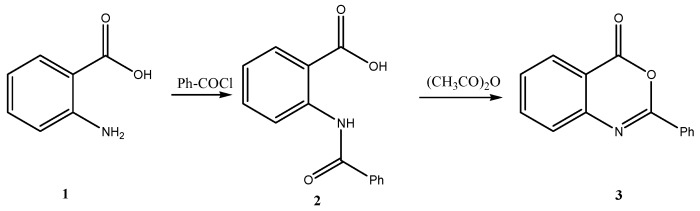
Synthesis of 2-phenyl-1,3-(4*H*)-benzoxazin-4-one **3**.

In the literature, the synthesis of 2-phenyl-1,3-(4*H*)-benzoxazin-4-one **3** (Scheme 1) was carried out from anthranilic acid **1** with benzoyl chloride via an intermediate **2** that cyclizes under the acetic anhydride action, at reflux heating [33]. In this work, we took it back by using reflux heating and microwave irradiation to highlight the efficiency of the latter. Thus, 97% of the product yield was obtained in a few minutes under microwave irradiation against 90% after 2 h of the conventional reflux heating method.

**Scheme 2:**
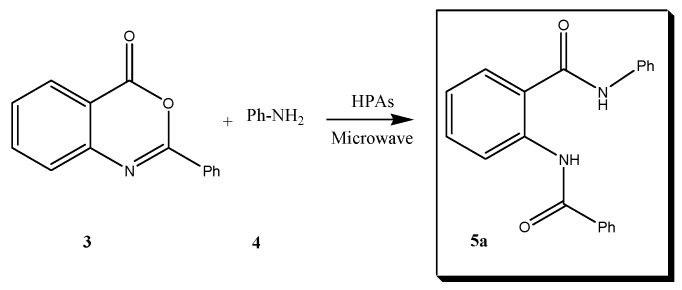
Synthesis of 2-benzoylamino-*N*-phenylbenzamide **5a** by condensation of 2-phenyl-1,3-(4*H*)-benzoxazin-4-one **3** and aniline **4** in the presence of HPAs under microwave irradiation in solvent-free conditions.

The 2-phenyl-1,3-(4*H*)-benzoxazin-4-one **3** compound was used for the 2-benzoylamino-*N*-phenylbenzamide **5a** synthesis from its condensation with aniline **4**. The reaction was conducted, under microwave irradiation, in solvent-free conditions, using a series of Keggin-type heteropolyacids, HnXM_12_O_40_ (abbreviated as XM_12_, where X = P or Si and M = W or Mo) (Scheme 2). Results are summarized in Table 1.

**Scheme 3:**
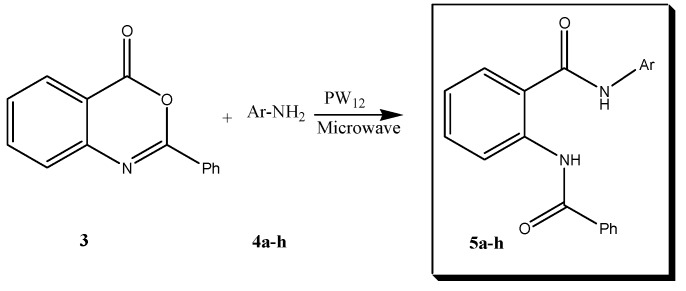
2-Benzoylamino-*N*-phenylbenzamide derivatives **5a**–**h** synthesis by condensation of 2-phenyl-1,3-(4*H*)-benzoxazin-4-one **3** with various substituted anilines **4a**–**h** in the presence of PW_12_ catalyst under microwave irradiation in solvent-free conditions.

2-Benzoylamino-*N*-phenylbenzamide yields (Table 1) depended on the nature of both the metal atom (W, Mo) and the heteroatom (P, Si) of HPA. Thus, W-based HPAs were more efficient than Mo-based (72–80% against 56–65% of **5a** yield). Phosphorus heteroatoms, which make the HPA more active, unlike siliceous heteroatoms, resulted in a yield of **5a** of 80% against 72% for W-based HPAs and 65% against 56% for Mo-based HPAs. The results obtained show that the decrease in yield (PW_12_ > SiW_12_ > PMo_12_ > SiMo_12_) follows that of the acidity strength [34]. Thus, PW_12_heteropolyacid was chosenas the catalyst to synthesize a series of 2-benzoylamino-*N*-phenylbenzamide derivatives **5a**–**h** with substituted anilines **4a**–**h** in the same conditions (Scheme 3). The products are obtained in a few minutes. The results are summarized in Table 2. 

The aniline substituent group nature shows a strong impact on the yields. Thus, the presence of electron donating groups led to a yield increase. With methyl and hydroxy groups in C_6_H_4_, the yields are 85% and 92%, respectively, against 80% for the phenyl. These groups are beneficial because of their high electron density, induced by the aromatic system unlike, the electron withdrawing group as chloro, which led to a yield decrease from 80% to 78%. The presence of a second chlorine atom in the aniline also led to a yield decrease from 78% to 65%. Among dichloroanilines, 2,4-dichloro-C_6_H_3_ gave the better yield (73% against 65–70%). This decrease is attributed to the group steric effect.

**Scheme 4:**
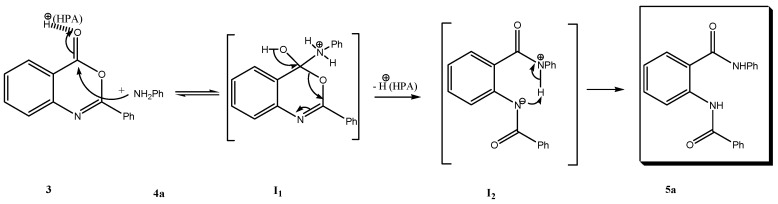
Proposed mechanism for the 2-benzoylamino-*N*-phenylbenzamide **5a** formation.

Scheme 4 shows a plausible mechanism of the 2-benzoylamino-*N*-phenylbenzamide **5a** formation in the heteropolyacid presence. The initial step corresponds to the protonation of carbonyl on a Brønsted site of HPA favoring the amine attack that leads to the intermediate **I_1_**. Thelatter is then deprotonated to give another intermediate **I_2_** and the released proton is then recovered by the HPA. Finally, a proton transfer from the aniline to the amide nitrogen takes place, thus leading to the final product. It is known that the presence of an electron donating group favors the amine basic character.

## 3. Antibacterial, Antifungal of the Synthesized Compounds

The synthesized compounds were screened for their antimicrobial activity against fungal and bacterial pathogenic strains by the disc diffusion method [35,36,37]. Gram-negative bacterial strains, namely *Escherichia coli* (ATCC-11105) and *Pseudomonas aeruginosa* (ATCC-9027), and Gram-positive bacteria, namely *Staphylococcus aureus* (ATCC-6538) and *Bacillus subtilis* (ATCC-6633), were chosen as model bacterial strains, and fungi, namely *Candida albicans* (ATCC-10231) and *Aspergillus brasiliensis* ATCC-16404)). Agar plates, containing 2-benzoylamino-*N*-phenylbenzamide products dissolved in dimethylsulfoxide (600 μg/mL) were inoculated uniformly from fresh bacterial culture and incubated at 37 °C for 24 h. Antimicrobial activity data are given in Table 3.

Antibacterial screening revealed that all tested compounds **5a**–**h** showed from moderate (++) to good (+++) inhibition against bacterial strains: *E. coli*, *P. aeruginosa*. For *S. aureus* and *B. subtilis* bacterial strains, **5a** and **5f**, respectively, do not show any antibacterial activity. Antifungal screening also revealed that all the tested compounds **5a**–**h** showed a good (+++) inhibition against *C. albicans* and *A. brasiliensis*.

The antibacterial and antifungal activities of a compound capable of inhibiting the visible growth of bacterial and fungal strains are defined by the value of the MIC that corresponds to its lower concentration. In order to determine the minimum inhibition concentration (MIC) values of the compound **5e** against the bacterial strains mentioned above, it was dissolved in DMSO at different concentrations (100, 200, 300, 400 and 600 μg/mL). The results are summarized in Table 4. The MIC values found for compound **5e** are less than 100 μg/mL for *E. coli*, *P. aeruginosa*, *B. subtilis*, and *C. albicans*, and they are 100–200 and 300–400 μg/mL for *S. aureus* and *A. brasiliensis*, respectively.

## 4. Conclusions

High 2-benzoylamino-*N*-phenylbenzamides derivatives **5a**–**h** yields (66–92%) with short reaction times (3 min) were obtained using a microwave irradiation and Keggin-type heteropolyacids as catalysts in solvent free conditions. A plausible mechanism of the 2-benzoylamino-*N*-phenylbenzamide **5a** formation was proposed. 2-Benzoylamino-*N*-phenyl benzamides derivatives **5a**–**h** showed both moderate and good antibacterial and antifungal activities. These results give an idea of further research on these molecules in the biological domain.

## 5. Experimental Section

### 5.1. General

Pure heteropolyacids H_n_XM_12_O_40_ (PM_12_) were prepared by the standard method involving the synthesis of the corresponding sodium salt and the extraction of acid by diethyl ether and its purification by crystallization in water at 4 °C [38].

All research chemicals and solvents were purchased from Sigma-Aldrich (Sigma-Aldrich, Saint-Quentin-Fallavier, France) and were used as such for the reactions. The progress of all the reactions was monitored by thin-layer chromatography (TLC) using glass plates precoated with silica gel-60 F254 to a thickness of 0.5 mm. The melting points were taken in an open capillary tube using an Electrothermal melting point apparatus (Electrotermal, Rochford, Great Britain). The values are reported in °C and are uncorrected. NMR spectra were recorded with a Bruker Avance 300 spectrometer (300 MHz (^1^H) and 75 MHz (^13^C)) (Bruker Biospin GmbH, Rheinstetten, Germany). Chemical shifts are expressed in parts per million (ppm) downfield from using tetramethylsilane (TMS). Data are reported as follows: chemical shift (multiplicity (s: singlet, d: doublet, dd: double doublet, ddd: double double doublet, dm: double multiplet, dt: double triplet, t: triplet, td triple doublet, tm, triple multiplet, tt: triple triplet, q: quartet, quint: quintuplet, m: multiplet, br: broad), coupling constants (*J*) in Hertz, integration). All the compounds gave satisfactory elemental analysis within ± 0.4% of theoretical values.

The multimode microwave reactor (a modified Candy MGA 20 M microwave oven) has a single magnetron (2450 MHz) with a maximum delivered power of 800 W. Experiments were carried out in a Pyrex reactor fitted with a condenser. During experiments, the temperature was monitored with an external infrared thermometer, Flashpoint FZ400 (Shenzhen Jumaoyuan Science and Technology CO., LTD, Guangdong, China). Our modifications to a domestic microwave oven, adopted since 1992, are similar to those described, currently, for microwave chemistry experiments [39]. In a typical design, a hole was drilled for a condenser tube in the oven top. External steel tube of the same diameter (~12 cm long) was welded to the hole in order to eliminate possible microwave leakage. The microwave equipment operates within the safety limits prescribed: the accepted limit on the safe stray leakage of the microwave power density is 10 mW/cm^2^ at 2450 MHz measured at a 50 mm distance from the equipment (microwave leakage detector). The apparatus has been adapted for laboratory applications with an external reflux condenser, multi-limb vacuum receivers, and a Dean Stark trap.

### 5.2. General Procedure for the Preparation of 2-Phenyl-3,1-(4H)-benzoxazin-4-one ***3***

*Method I* (conventional heating): A mixture of anthranilic acid (10 mmol) and benzoyl chloride (10 mmol) was carried out under reflux in toluene (15 mL) for 2 h. A white solid wasobtained. The latter wasthen treated with the acetic anhydride under reflux for 2 h.

*Method II* (microwave irradiation): A mixture of anthranilic acid (10 mmol) and benzoyl chloride (10 mmol) and 10 mL of toluene was carried out under microwave irradiation. The power was initially set to 420 W for 5 min, and then it was increased to 510 W for 7 min. A white solid wasobtained. The latter with the acetic anhydride (10 mL) irradiated under microwave at 500 W for 8 min. The obtained solid was washed by the water to eliminate acid.

2-Phenyl-3,1-(4*H*)-benzoxazin-4-one (**3**). White solid, Yield 97%; m.p. 126 °C; ^1^H-NMR (CDCl_3_, 300 MHz): δ = 7.24–8.35 (m, 9H, Ar-H) ppm; ^13^C-NMR (CDCl_3_, 75 MHz): δ = 116.62, 126.80, 127.73, 128.02, 128.26, 129.81, 132.13, 135.96, 146.46, 156.52, 158.80 ppm; Anal. Calcd. for C_14_H_9_NO_2_: C, 75.58; H, 4.12; N, 6.28;O, 14.00. Found: C, 75.33; H, 4.06; N, 6.27; O, 14.33%.

### 5.3. General Procedure for the Preparation of 2-Benzoylamino-N-phenylbenzamide Derivatives ***5a**–**h***

To a mixture of 2-phenyl-3,1-(4*H*)-benzoxazin-4-one (10 mmol) and amines (10 mmol) was added the catalyst heteropolyacid (1.2 mol %). This mixture was heated by microwave, initially set to 300 W for 3 min and then it was increased to 450 W for 10 min. The obtained solid was washed by the water to eliminate acid. The ^1^H-NMR and ^13^C-NMR spectrums of compounds **5a**–**h** in Supplementary Materials.

*2-Benzoylamino-N-phenylbenzamide* (**5a**): Yield 80%; m.p. 281 °C; ^1^H-NMR (DMSO-*d*_6_, 300.13 MHz): δ = 11.68 (s, 1H, NH), 10.55 (s, 1H, NH), 8.47 (d, *J* = 8.6 Hz, 1H), 7.92 (d, *J* = 8.1 Hz, 3H), 7.72 (d, *J* = 7.56 Hz, 2H), 7.65–7.69 (m, 4H), 7.30–7.40 (m, 3H), 7.16(t, *J* = 7.02 Hz, 1H); ^13^C-NMR (DMSO-*d*_6_, 75.47MHz): δ = 166.90, 166.85, 138.16, 137.98, 133.97, 133.90, 131.77, 131.56, 128.44, 128.18, 126.52, 122.82, 120.67 ppm. Anal. Calcd. for C_20_H_16_N_2_O_2_: C, 76.13; H, 5.25; N, 8.98; O, 9.63. Found: C, 75.93; H, 5.10; N, 8.86; O, 10.11%.

*2-Benzoylamino-N-(4-methylphenyl)benzamide* (**5b**): Yield 85%; m.p. 123 °C; ^1^H-NMR (DMSO-*d*_6_, 300.13 MHz): δ = 11.81 (s, 1H, NH), 10.49 (s, 1H, NH), 8.63 (d, *J* = 8.3Hz, 1H), 8.54 (d, *J* = 9 Hz, 3H), 7.61–7.32 (m, 6H), 7.10–7.20 (m, 3H), 2.29 (s, 3H); ^13^C-NMR (DMSO-*d*_6_, 75.47 MHz): δ = 167.80, 165.00, 139.31, 136.34, 133.88, 132.50, 131.77, 129.62, 129.38, 128.04, 127.46, 123.65, 121.67, 121.56, 21.10 ppm. Calcd. for C_21_H_18_N_2_O_2_: C, 76.55; H, 5.60; N, 8.53; O, 9.31. Found: C, 76.43; H, 5.49; N, 8.48; O, 9.69%.

*2-Benzoylamino-N-(4-hydroxyphenyl)benzamide* (**5c**): Yield 92%; m.p. 160 °C; ^1^H-NMR (DMSO-*d*_6_, 300.13 MHz): δ = 11.99 (s, 1H, NH), 10.37 (s, 1H, NH), 9.38 (s, 1H), 8.57 (d, *J* = 8.4 Hz, 1H), 8.23 (d, *J* = 8.4 Hz, 1H), 7.65 (dd, *J* = 8.4 Hz, 4H), 7.63–7.47 (m,4H), 7.47 (d, *J* = 8.4 Hz, 2H), 7.16 (d, *J* = 6 Hz, 1H); ^13^C-NMR (DMSO-*d*_6_, 75.47 MHz): δ = 166.90, 166.85, 152.16, 137.98, 133.97, 133.90, 131.77, 131.56, 127.54, 127.28, 125.52, 121.82, 120.67 ppm. Calcd. for C_20_H_16_N_2_O_3_: C, 72.50; H, 4.96; N, 8.49; O, 14.04. Found: C, 72.28; H, 4.85; N, 8.43; O, 14.44%.

*2-Benzoylamino-N-(4-chlorophenyl)benzamide* (**5d**): Yield 78%; m.p. 161 °C; ^1^H-NMR (DMSO-*d*_6_, 300.13 MHz): δ = 11.56 (s, 1H, NH), 10.66 (s, 1H, NH), 8.44 (d, *J* = 8.2 Hz, 1H), 7.92 (d, *J* = 7.1 Hz, 3H), 7.74 (d, *J* = 8.6 Hz, 2H), 7.65–7.57 (m, 4H), 7.42 (d, *J* = 9.1 Hz, 2H), 7.26 (t, *J* = 9.1 Hz, 1H); ^13^C-NMR (DMSO-*d*_6_, 75.47 MHz): δ = 167.48, 164.69, 138.62, 137.57, 134.50,132.35, 132.05, 129.05, 128.89, 128.60, 127.95, 127.09, 123.37, 122.97, 122.59, 121.52 ppm. Calcd. for C_20_H_15_ClN_2_O_2_: C, 68.55; H, 4.36; N,10.13; O, 16.95. Found: C, 68.48; H, 4.31; Cl, 10.11; N, 7.99; O, 9.12%.

*2-Benzoylamino-N-(2,4-dichlorophenyl)benzamide* (**5e**): Yield 73%; m.p. 140 °C; ^1^H-NMR (DMSO-*d*_6_, 300.13 MHz): δ = 11.93 (s, 1H, NH),10.49 (s, 1H, NH), 8.58 (d, *J* = 8.6 Hz, 1H), 8.04 (d, *J* = 6.75 Hz, 3H), 7.72 (s, 1H), 7.65–7.49 (m, 6H), 7.43 (t, *J* = 8.1 Hz, 1H) ppm. ^13^C-NMR (DMSO-*d*_6_, 75.47 MHz): δ = 164.10, 157.65, 147.39, 136.47, 134.90, 134.10, 132.13, 131.98, 131.18, 130.51, 129.16, 127.53, 127.43, 127.40, 127.03, 125.86, 125.68, 120.22 ppm. Calcd. for C_20_H_14_Cl_2_N_2_O_2_: C, 62.50; H, 3.71; Cl, 18.48; N, 7.37; O, 7.94 Found: C, 62.34; H, 3.66; Cl, 18.41; N, 7.27; O, 8.31%.

*2-Benzoylamino-N-(2,5-dichlorophenyl)benzamide* (**5f**): Yield 68%; m.p. 167 °C; ^1^H-NMR (DMSO-*d*_6_, 300.13 MHz): δ = 11.75 (s, 1H, NH), 10.45 (s, 1H, NH), 8.49 (d, *J* = 8.4 Hz, 1H), 7.91 (d, *J* = 8.0 Hz, 2H), 7.73 (s, 1H), 7.67 (d, *J* = 7.29 Hz, 1H), 7.61–7.40 (m, 7H), 7.41 (t, *J* = 8.1 Hz, 1H) ppm, ^13^C-NMR (DMSO-*d*_6_, 75.47 MHz): δ = 167.98, 165.30, 139.21, 138.77, 134.90, 132.81, 132.45, 131.35, 130.96, 129.72, 129.41, 127.55, 126.15, 123.98, 123.90, 122.53, 122.34, 121.90 ppm. Calcd. For C_20_H_14_Cl_2_N_2_O_2_: C, 62.50; H, 3.71; Cl, 18.48; N, 7.37; O, 7.94 Found: C, 62.34; H, 3.66; Cl, 18.41; N, 7.27; O, 8.31%.

*2-Benzoylamino-N-(2,6-dichlorophenyl)benzamide* (**5g**): Yield 65%; m.p. 162 °C; ^1^H-NMR (DMSO-*d*_6_, 300.13 MHz): δ = 12.08 (s, 1H, NH), 10.75 (s, 1H, NH), 8.70 (d, *J* = 8.2 Hz, 1H), 8.11 (d, *J* = 6.9 Hz, 2H), 7.87 (d, *J* = 6.9 Hz, 1H), 7.62–7.50 (m, 5H), 7.45 (d, *J* = 6.9 Hz, 2H), 7.32 (t, *J* = 8.1 Hz, 1H) ppm; ^13^C-NMR (DMSO-*d*_6_, 75.47 MHz): δ = 168.38, 165.00, 140.11, 134.74, 134.52, 133.67, 132.90,132.63, 130.32, 129.47, 129.15, 127.32, 123.66, 121.18, 119.97 ppm. Calcd. for C_20_H_14_Cl_2_N_2_O_2_: C, 62.50; H, 3.71; Cl, 18.48; N, 7.37; O, 7.94 Found: C, 62.34; H, 3.66; Cl, 18.41; N, 7.27; O, 8.31%.

*2-Benzoylamino-N-(3,4-dichlorophenyl)benzamide* (**5h**): Yield 70%; m.p. 192 °C; ^1^H-NMR (DMSO-*d*_6_, 300.13 MHz): δ = 11.36 (s, 1H, NH), 10.71 (s, 1H, NH), 8.34 (d, *J* = 7.29 Hz, 1H), 8.01 (s, 1H), 7.92–7.85 (m, 3H), 7.70–7.31 (m, 5H), 7.28 (t, *J* = 6.75Hz, 1H) ppm; ^13^C-NMR (DMSO-*d*_6_, 75.47 MHz): δ = 167.98, 165.30, 139.23, 138.77, 134.90, 132.81, 132.45, 131.35, 130.96, 129.41, 129.27, 126.15, 123.99, 123.91, 122.54, 122.35, 121.30 ppm. Calcd. for C_20_H_14_Cl_2_N_2_O_2_: C, 62.50; H, 3.71; Cl, 18.48; N, 7.37; O, 7.94 Found: C, 62.34; H, 3.66;Cl, 18.41; N, 7.27; O, 8.31%.

### 5.4. Screening for Antibacterial Activity by the Agar Diffusion Method for 2-Benzoylamino-N-phenylbenzamide Derivatives ***5a**–**h***

The antimicrobial activities of compounds **5a**–**h** were evaluated for their antibacterial activities against *S. aureus* (ATCC29213), *B. subtilis* (ATCC6633), *E. coli* (ATCC11105)), *P. aeruginosa* (ATCC9027), and *Bacillus subtilis* (ATCC-6633) bacterial strains and their anti-fungal activities against *C. albicans* (ATCC-10231) and *A. brasiliensis* (ATCC-16404) by the agar diffusion method [37]. A sterile physiological water solution contained a bacterial colonies, was prepared at room temperature, with an optical density of 0.08–0.10 corresponding to a concentration of 10^6^ cells/mL. The bacterial solution was inoculated in the Muller-Hinton agar medium by swabbing using Petri dishes at room temperature. The tested compounds were dissolved in dimethylsulfoxide (DMSO) with a concentration of 600 μg/mL. Twenty-five microlliters of tested sample were poured onto filter paper discs 6 mm in diameter, which were then delicately placed on the surface of the agar plates. These were later maintained at 37 °C for 24 h. Activities were determined by measuring the diameter of the inhibition zone (mm).

### 5.5. Minimum Inhibitory Concentration Determination of the Compound ***5e***

In order to determine the minimum inhibition concentration (MIC) values of the compound **5e**, different concentrations (100, 200, 300, 400 and 600 μg/mL)were considered. The MIC of the sample showedno turbidity and was recorded as the lowest concentration of the compound that would completely inhibit bacterial growth. Each test was performed in triplicate.

## Figures and Tables

**Table 1:** 2-Benzoylamino-*N*-phenylbenzamide yields (%).

Catalysts	PW_12_	SiW_12_	PMo_12_	SiMo_12_
Yields (%)	80	72	65	56

**Table 2:** Impact of aniline structure on reaction yield.

Products	ArNH_2_ (4a–h)	Yield (%)	M.p. (°C)	T (°C) ^a^
**5a**	C_6_H_5_	80	281–282	151
**5b**	4-Me-C_6_H_4_	85	123–124	155
**5c**	4-OH-C_6_H_4_	91	160–163	155
**5d**	4-Cl-C_6_H_4_	77	161–162	160
**5e**	2,4-Cl_2_-C_6_H_3_	73	140–142	106
**5f**	2,5-Cl_2_-C_6_H_3_	67	167–168	105
**5g**	2,6-Cl_2_-C_6_H_3_	65	162–164	121
**5h**	3,4-Cl_2_-C_6_H_3_	70	192–193	124

^a^ Temperature measurement by IR-thermometer.

**Table 3:** Antimicrobial activity data of the synthesized compounds **5a**–**h**, determined by the agar diffusion method.

Compounds	Bacteria				Fungi	
	*E. coli*	*S. aureus*	*P. aeruginosa*	*B. subtilis*	*C. albicans*	*A. brasiliensis*
**5a**	++	-	+++	+	+++	+++
**5b**	+	+	++	++	+++	+++
**5c**	++	++	++	++	+++	+++
**5d**	++	+++	++	+++	+++	+++
**5e**	++	+++	+++	+++	+++	+++
**5f**	++	+	++	-	+++	+++
**5g**	++	++	++	++	+++	+++
**5h**	++	+	++	++	+++	+++

The sensitivity of microorganisms, toward tested compounds, was identified in the following manner: no activity (- ≤ 8 mm), slightly active (8 < + < 16 mm), moderately active (16 ≤ ++ ≤ 20 mm) and highly active (+++ > 20 mm).

**Table 4:** Minimum inhibitory concentration (MIC) values of compound **5e**.

Concentration (µg/mL)	Bacteria				Fungi	
	*E. coli*	*S. aureus*	*P. aeruginosa*	*B. subtilis*	*C. albicans*	*A. brasiliensis*
600	++	+	+	++	+++	+++
400	+	+	+	++	+++	+
300	+	+	+	+	+++	-
200	+	+	+	+	++	-
100	+	-	+	+	++	-
MIC	≤100	100–200	≤100	≤100	≤100	300–400

The sensitivity of microorganisms, toward tested compounds, was identified in the following manner: no activity (- ≤ 8 mm), slightly active (8 < + < 16 mm), moderately active (16 ≤ ++ ≤ 20 mm), and highly active (+++ > 20 mm).

## Supplementary Materials

The ^1^H-NMR and ^13^C-NMR spectrums of compounds **5a**–**h** are available online.
