# Supplementary material for: Recyclable Keggin Heteropolyacids as an Environmentally Benign Catalyst for the Synthesis of New 2-Benzoylamino-N-phenyl-benzamide Derivatives under Microwave Irradiations at Solvent-Free Conditions and the Evaluation of Biological Activity
Source: Molecules. 2017 Dec 21;23(1):8. doi: 10.3390/molecules23010008 (PMC5943967; doi:10.3390/molecules23010008)
Supplement: Supplementary file 1 [file molecules-23-00008-s001.pdf]

Recyclable Keggin Heteropolyacids as an Environmentally Benign Catalyst for the Synthesis of New 2-Benzoylamino-*N*-phenyl-benzamide Derivatives under Microwave Irradiations at Solvent-Free Conditions and Evaluation of Biological Activity

Karima Ighilahriz-Boubchir[a,b], Baya Boutemeur-Kheddis[a], Cherifa Rabia[c], Malika Makhloufi-Chebli, Maamar Hamdi[a], Artur M.S. Silva[d]

[a] *Laboratoire de Chimie Organique Appliquée (Equipe Hétérocycles). Faculté de Chimie. Université des Sciences et de la Technologie Houari Boumediène. BP 32, El-Alia. 16111 Bab-Ezzouar. Algiers, Algeria*

[b]*Laboratoire de Physique et Chimie des Matériaux (LPCM), Université Mouloud Mammeri, BP17RP, 15000, Tizi Ouzou, Algeria*

[c] *Laboratoire de Chimie du Gaz Naturel. Faculté de Chimie. Université des Sciences et de la Technologie Houari Boumediène. BP 32, El -Alia. 16111 Bab-Ezzouar.Algiers, Algeria*

[d]*Department of Chemistry & QOPNA, University of Aveiro, 3810-193 Aveiro, Portugal*
Correspondence: bayakheddis@hotmail.com

Supporting Information

Table of Contents:

Figure S1. ^{1}H NMR spectrum of compound **5a** in DMSO (300.13 MHz)
Figure S2. ^{13}C NMR spectrum of compound **5a** in DMSO (75.47 MHz)

Figure S3. ^{1}H NMR spectrum of compound **5b** in DMSO (300.13 MHz)
Figure S4. ^{13}C NMR spectrum of compound **5b** in DMSO (75.47 MHz)

Figure S5. ^{1}H NMR spectrum of compound **5c** in DMSO (300.13 MHz)
Figure S6. ^{13}C NMR spectrum of compound **5c** in DMSO (75.47 MHz)

Figure S7. ^{1}H NMR spectrum of compound **5d** in DMSO (300.13 MHz)
Figure S8. ^{13}C NMR spectrum of compound **5d** in DMSO (75.47 MHz)

Figure S9. ^{1}H NMR spectrum of compound **5e** in DMSO (300.13 MHz)
Figure S10. ^{13}C NMR spectrum of compound **5e** in DMSO (75.47 MHz)

Figure S11. ^{1}H NMR spectrum of compound **5f** in DMSO (300.13 MHz)
Figure S12. ^{13}C NMR spectrum of compound **5f** in DMSO (75.47 MHz)

Figure S13. ^{1}H NMR spectrum of compound **5g** in DMSO (300.13 MHz)
Figure S14. ^{13}C NMR spectrum of compound **5g** in DMSO (75.47 MHz)

Figure S15. ^{1}H NMR spectrum of compound **5h** in DMSO (300.13 MHz)
Figure S16. ^{13}C NMR spectrum of compound **5h** in DMSO (75.47 MHz)

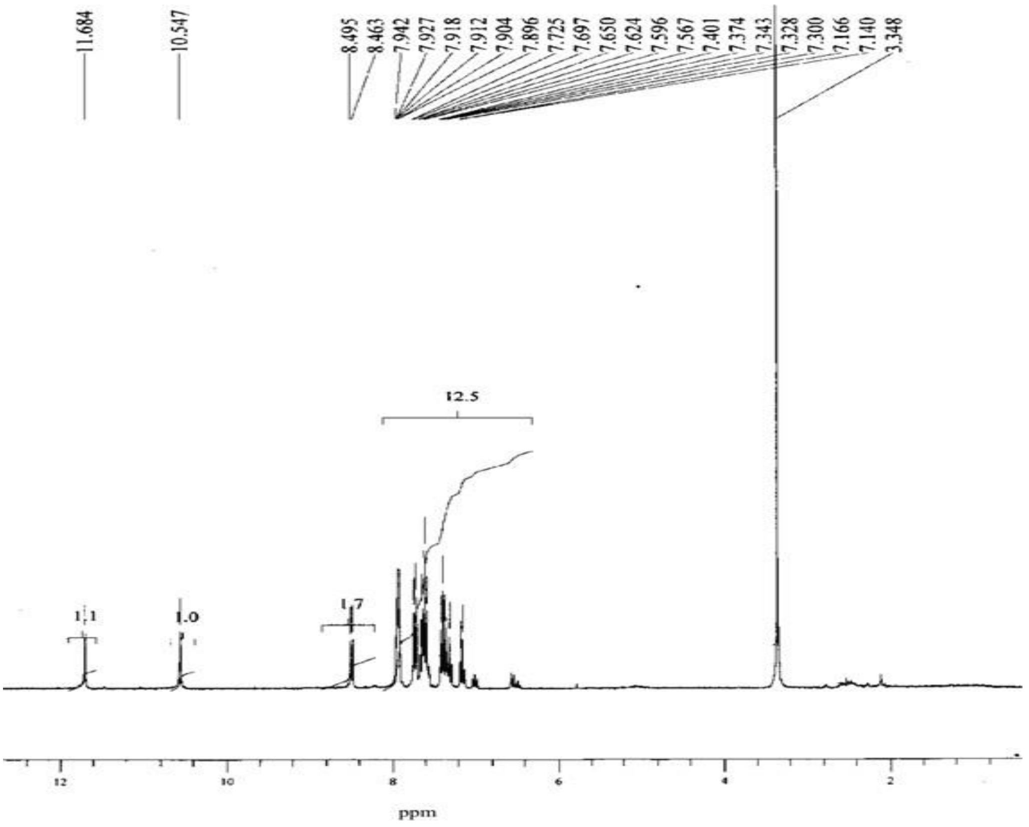

Figure S1. ^{1}H NMR spectrum of compound **5a** in DMSO (300.13 MHz)

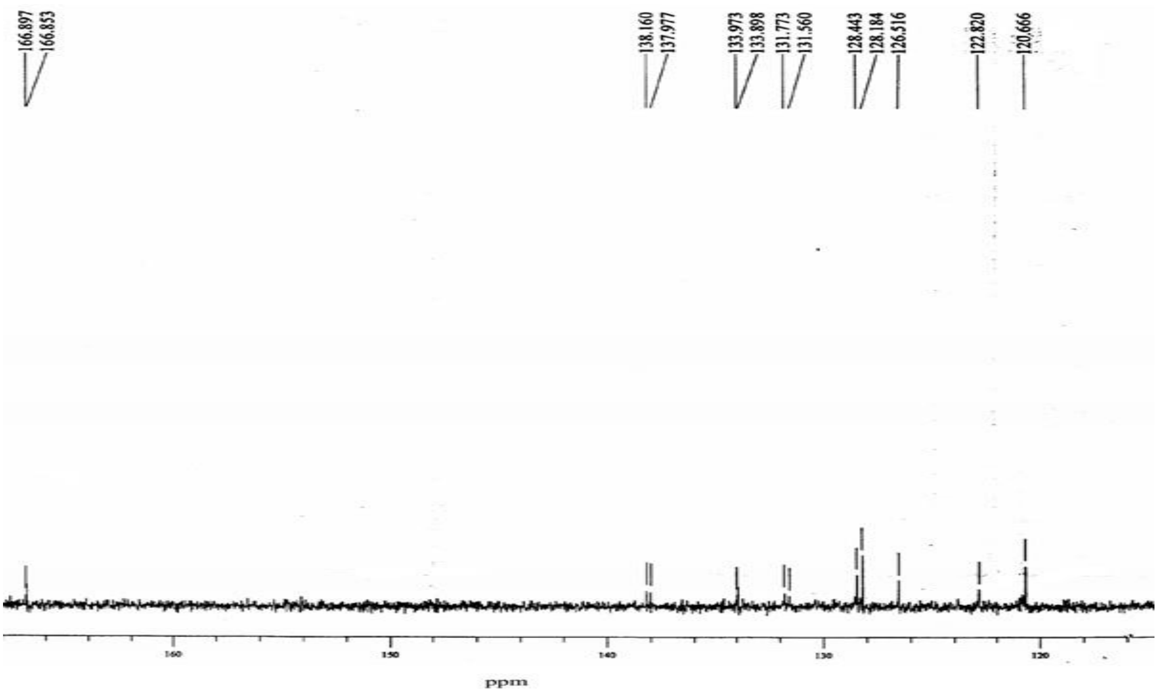

Figure S2. ^{13}C NMR spectrum of compound **5a** in DMSO (75.47 MHz)

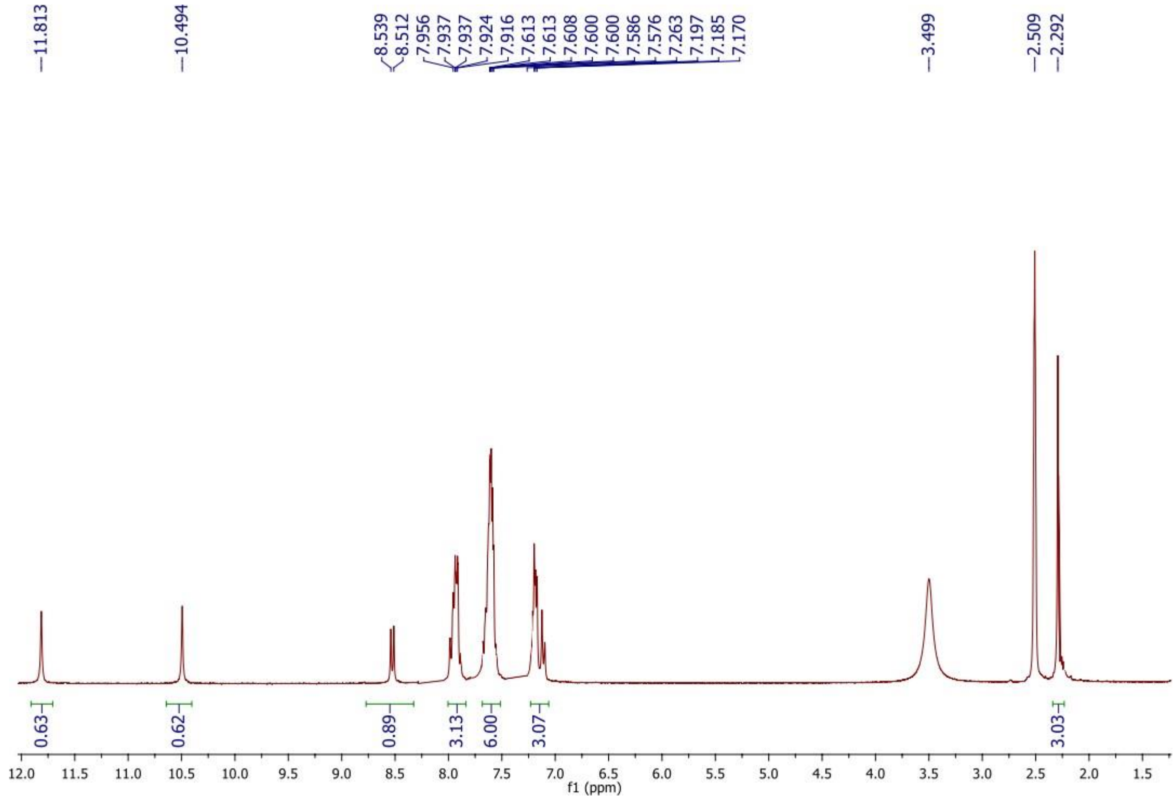

Figure S3. ^{1}H NMR spectrum of compound **5b** in DMSO (300.13 MHz)

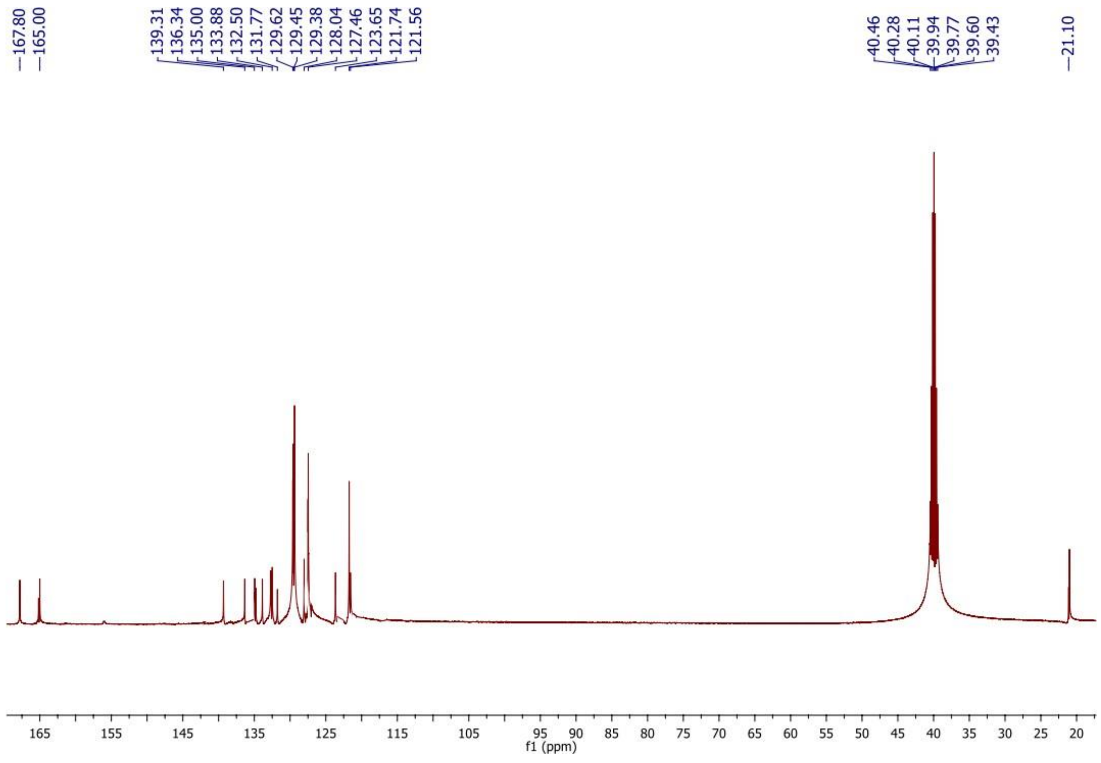

Figure S4. ^{13}C NMR spectrum of compound **5b** in DMSO (75.47 MHz)

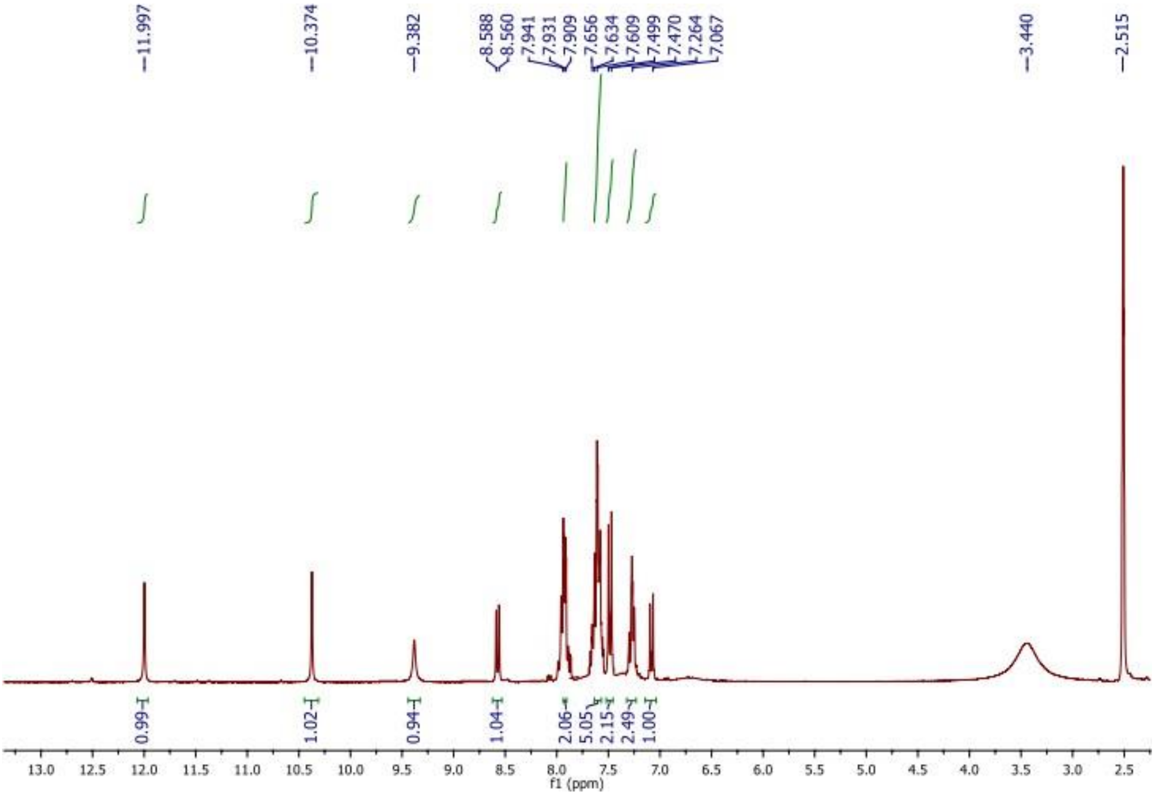

Figure S5. ^{1}H NMR spectrum of compound **5c** in DMSO (300.13 MHz)

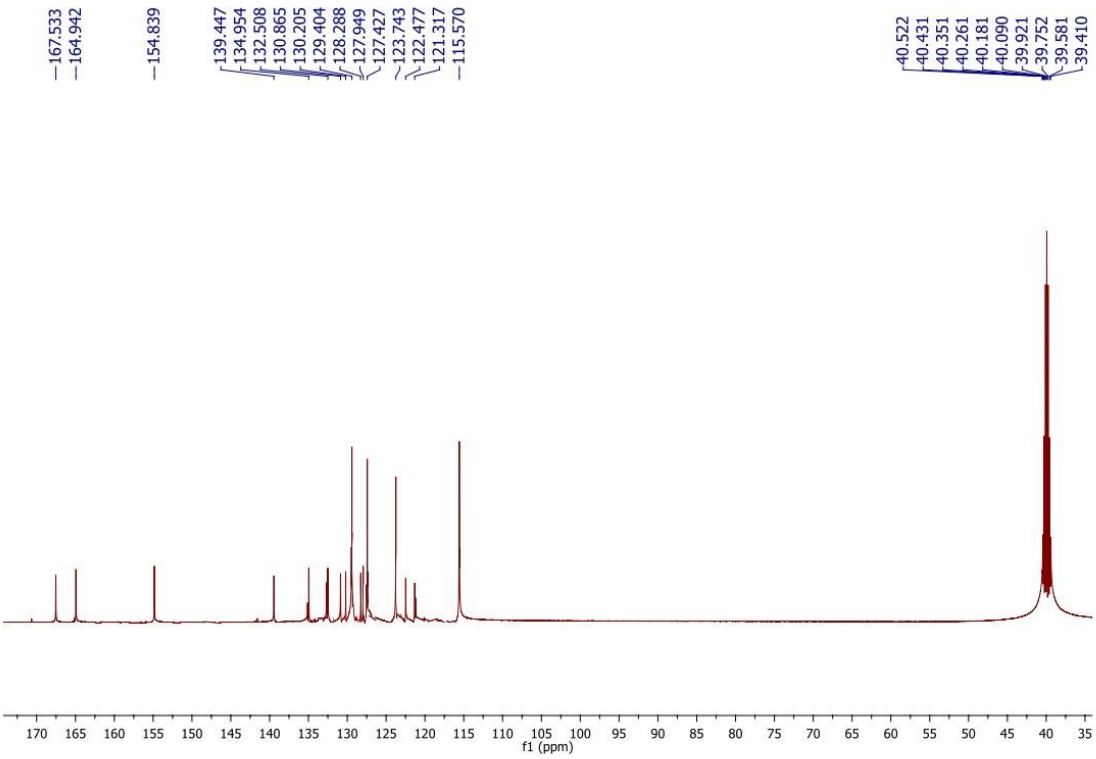

Figure S6. ^{13}C NMR spectrum of compound **5c** in DMSO (75.47 MHz)

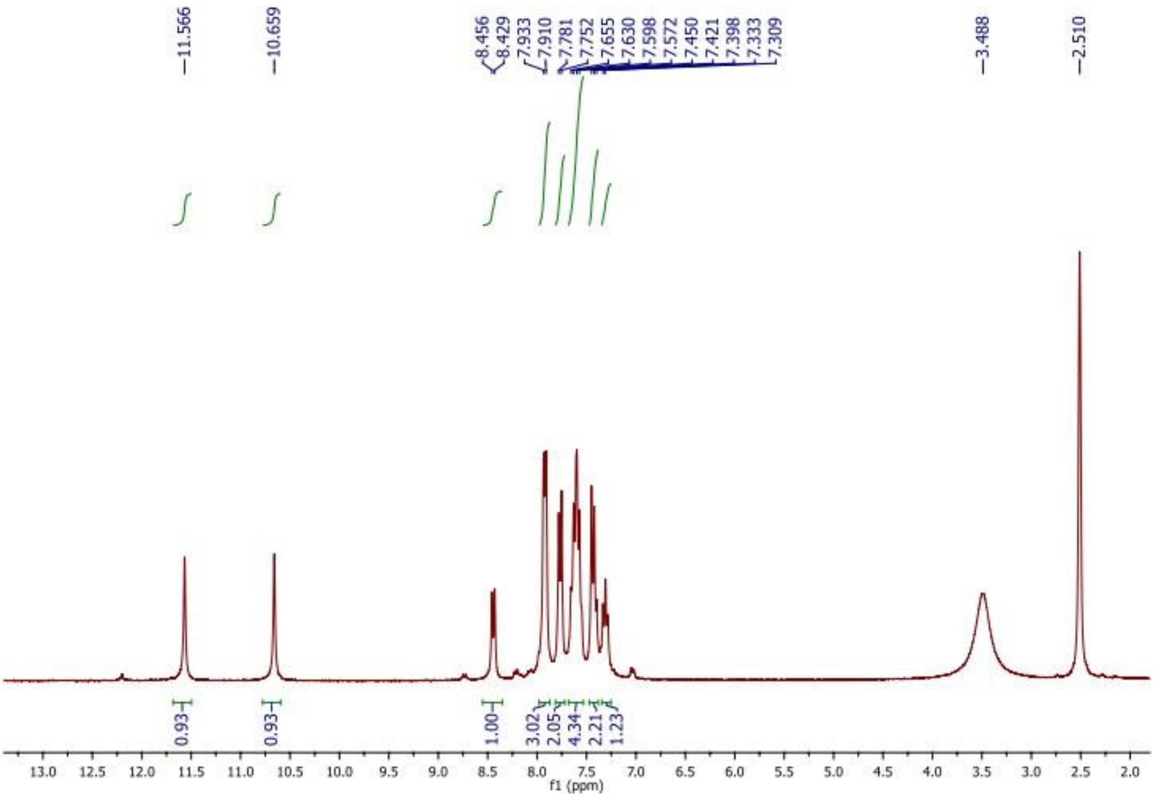

Figure S7. ^{1}H NMR spectrum of compound **5d** in DMSO (300.13 MHz)

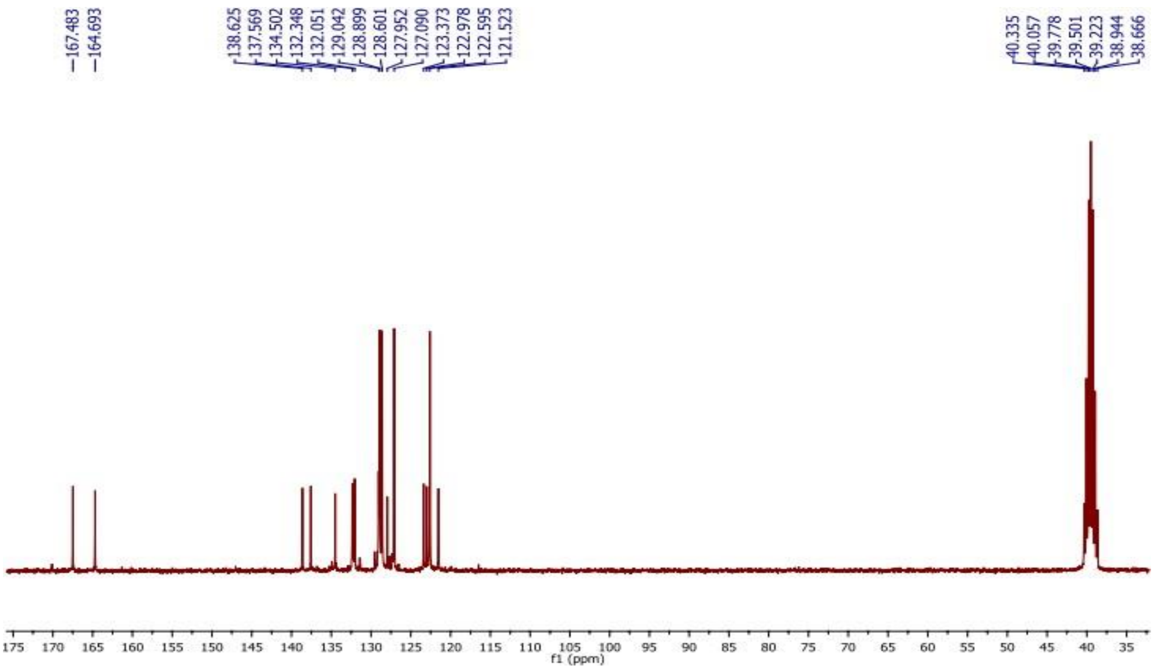

Figure S8. ^{13}C NMR spectrum of compound **5d** in DMSO (75.47 MHz)

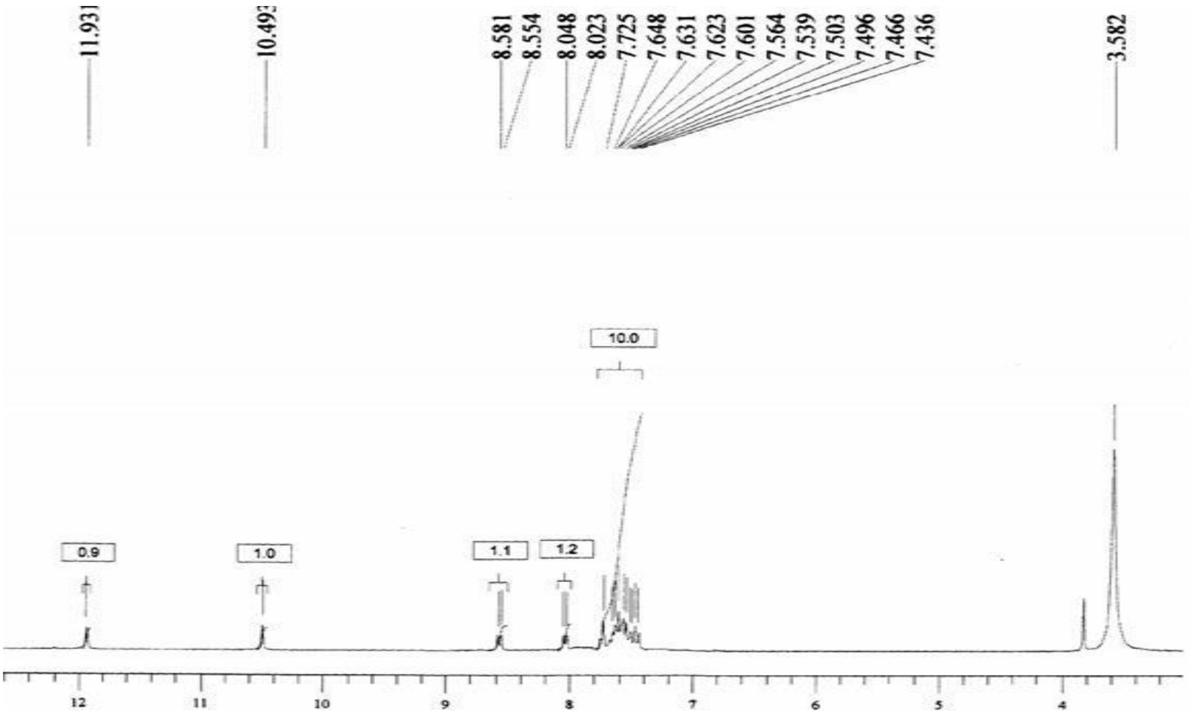

Figure S9. ^{1}H NMR spectrum of compound **5e** in DMSO (300.13 MHz)

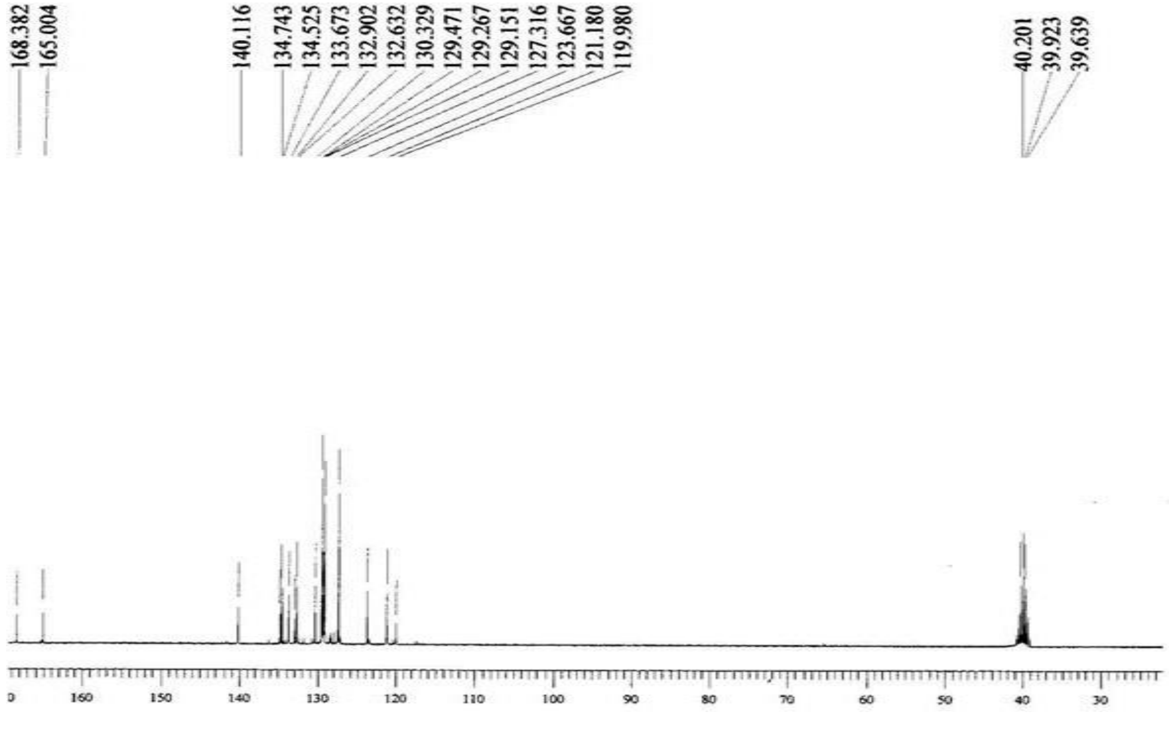

Figure S10. ^{13}C NMR spectrum of compound **5e** in DMSO (75.47 MHz)

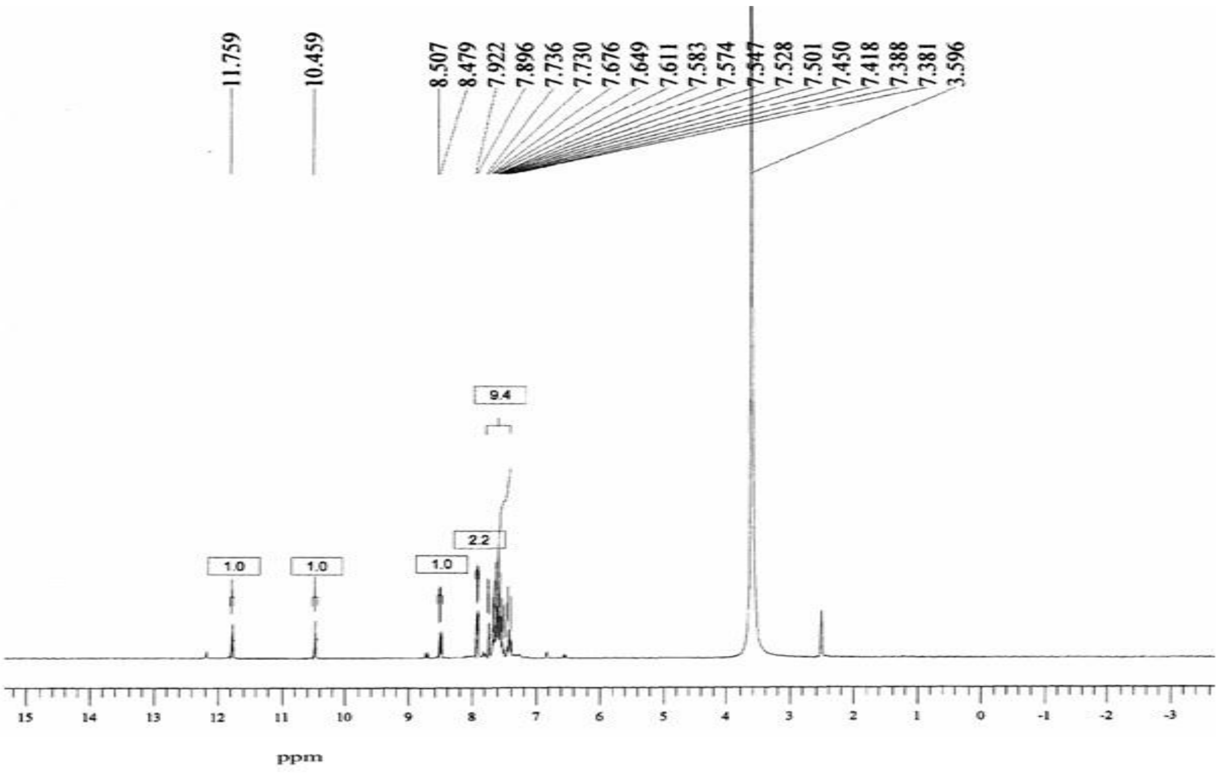

Figure S11. ^{1}H NMR spectrum of compound **5f** in DMSO (300.13 MHz)

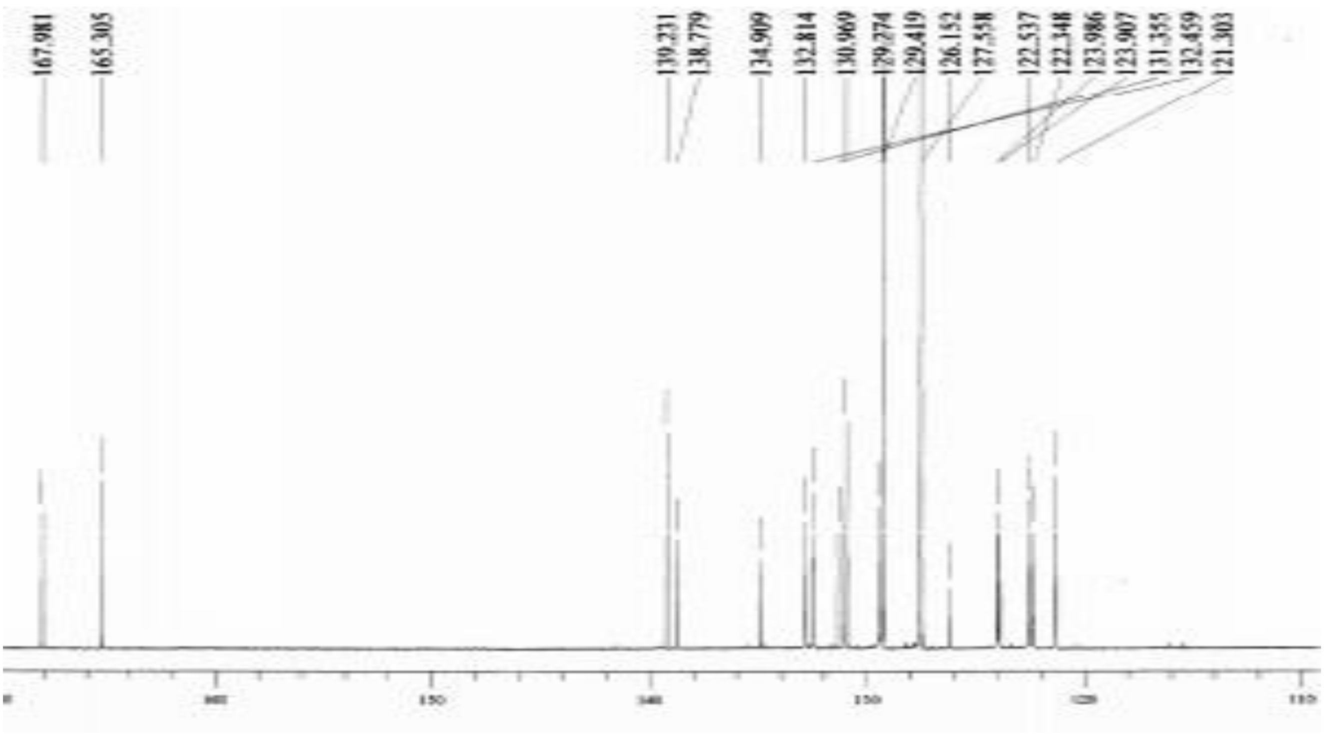

Figure S12. ^{13}C NMR spectrum of compound **5f** in DMSO (75.47 MHz)

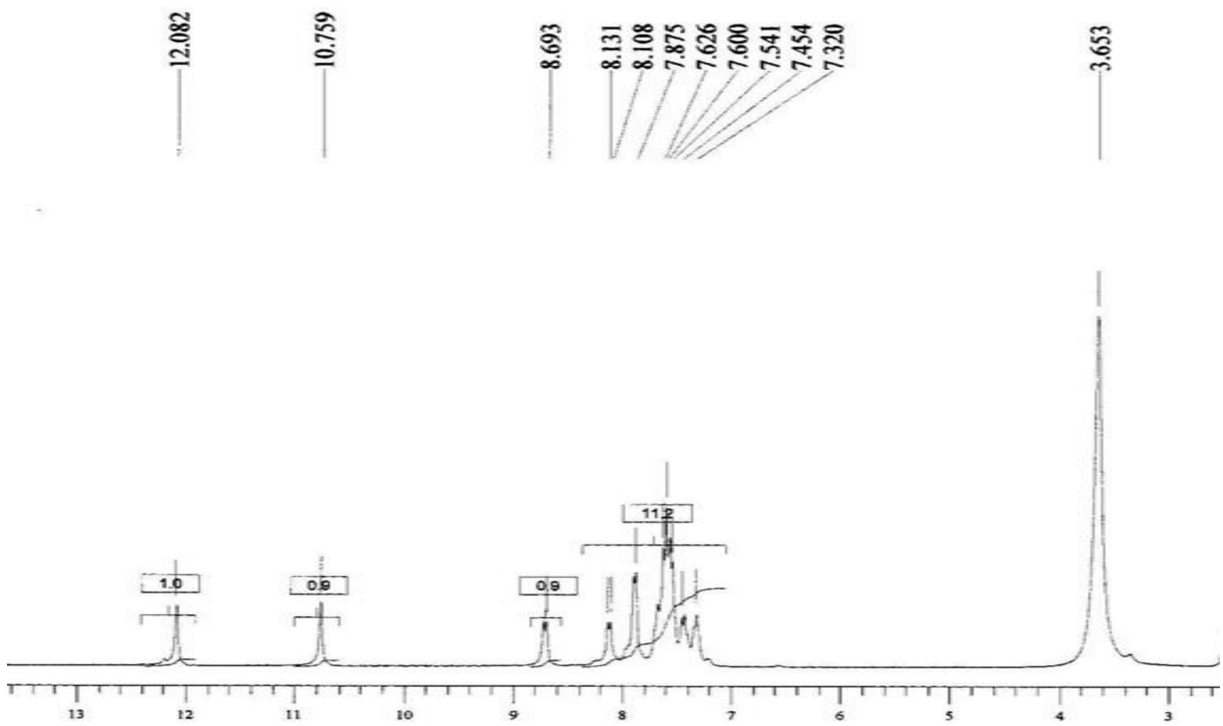

Figure S13. ^{1}H NMR spectrum of compound **5g** in DMSO (300.13 MHz)

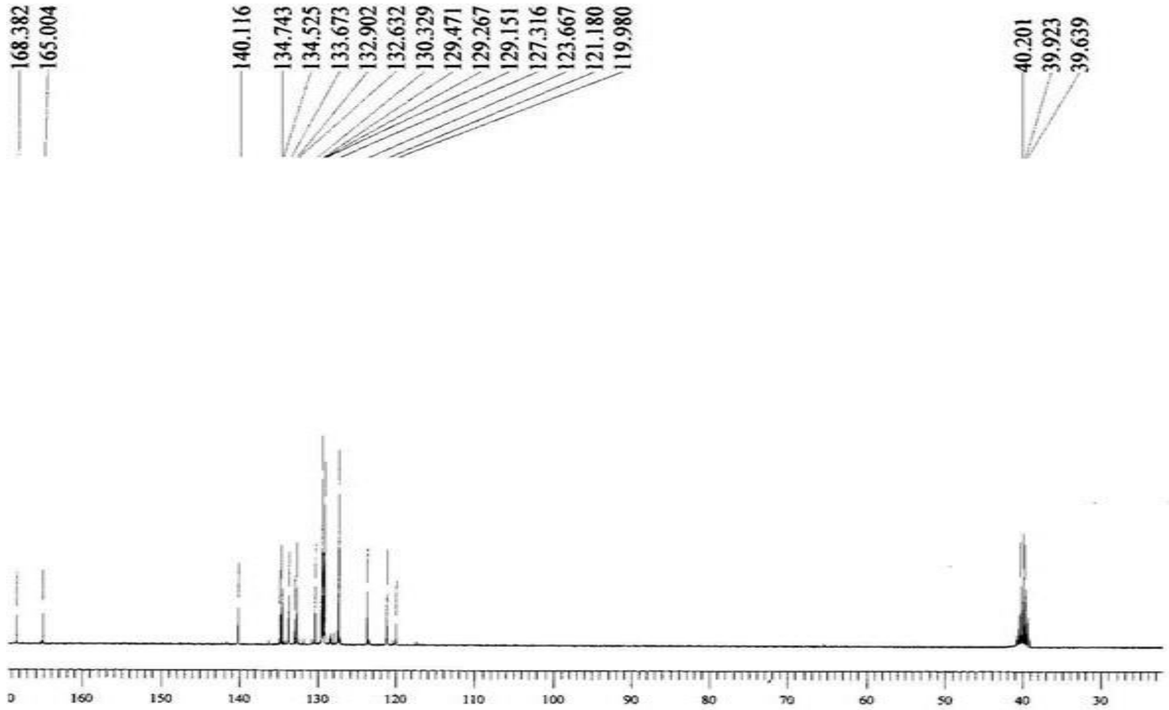

Figure S14. ^{13}C NMR spectrum of compound **5g** in DMSO (75.47 MHz)

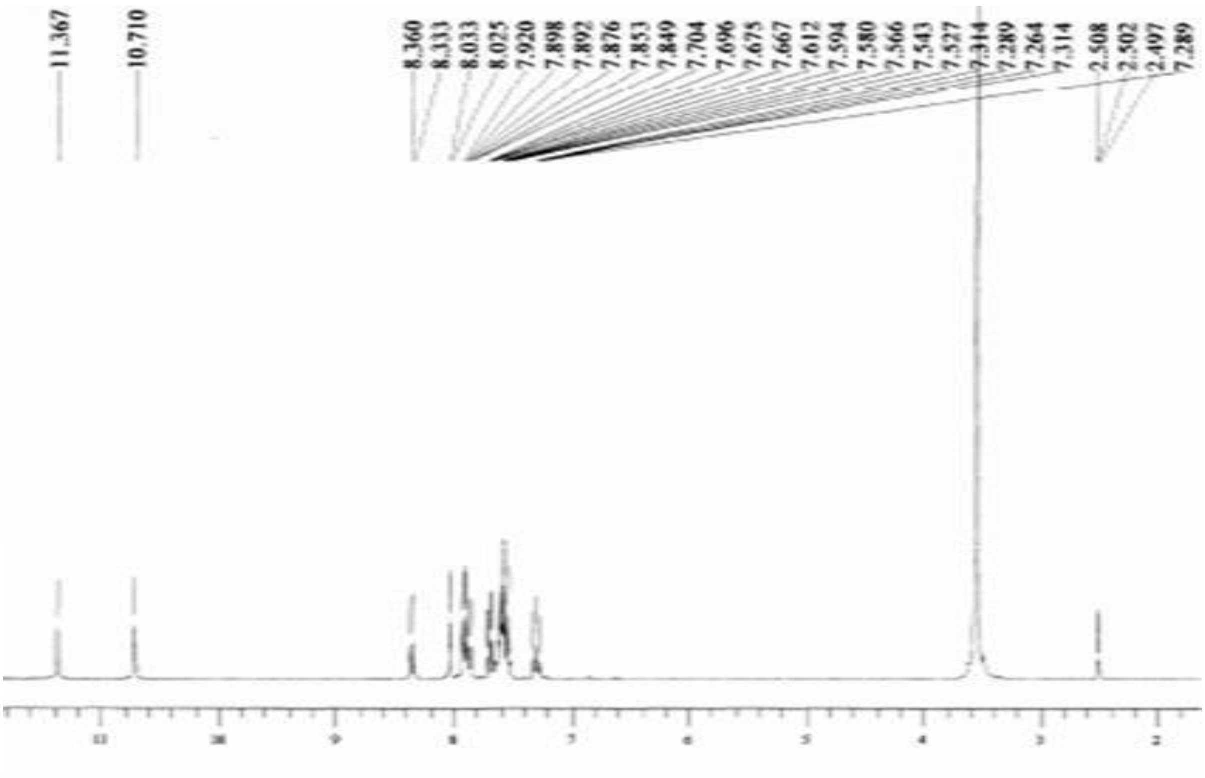

Figure S15. ^{1}H NMR spectrum of compound **5h** in DMSO (300.13 MHz)

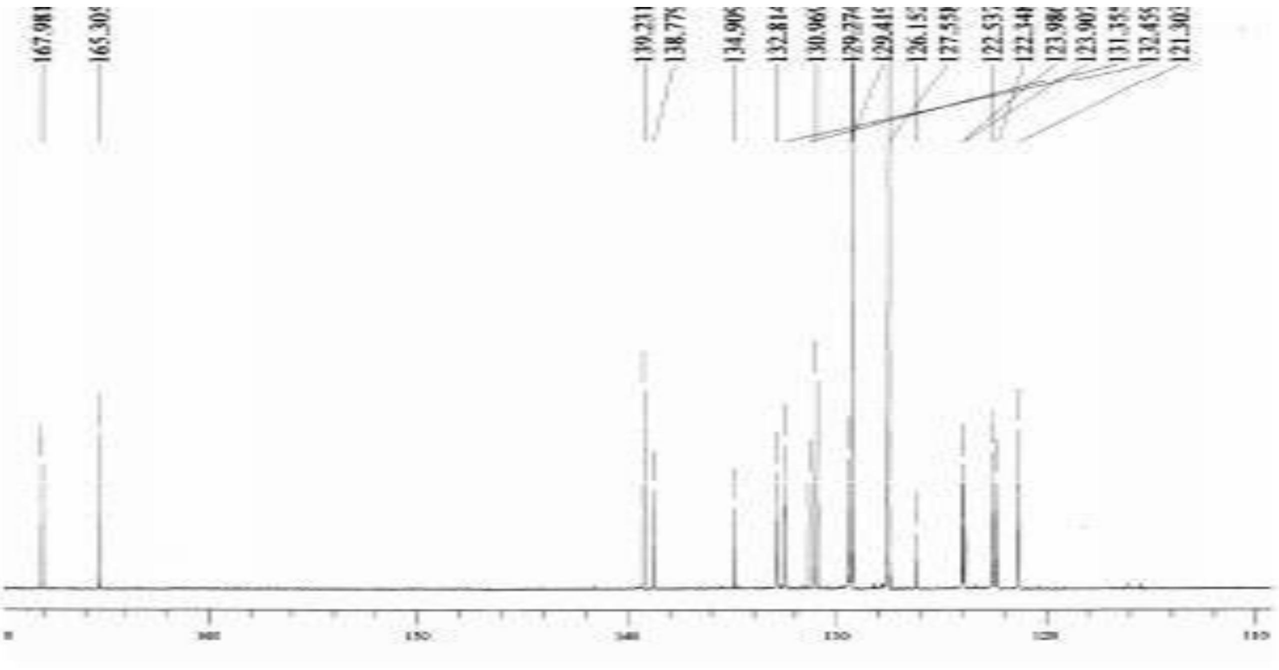

Figure S16. ^{13}C NMR spectrum of compound **5h** in DMSO (75.47 MHz)
